# Diabetic Cardiomyopathy and Ischemic Heart Disease: Prevention and Therapy by Exercise and Conditioning

**DOI:** 10.3390/ijms21082896

**Published:** 2020-04-21

**Authors:** Antonio Crisafulli, Pasquale Pagliaro, Silvana Roberto, Lucia Cugusi, Giuseppe Mercuro, Antigone Lazou, Christophe Beauloye, Luc Bertrand, Derek J. Hausenloy, Manuela Aragno, Claudia Penna

**Affiliations:** 1 Department of Medical Sciences and Public Health, University of Cagliari, 09124 Cagliari, Italy; crisafulli@tiscali.it (A.C.); silvy_rob@yahoo.it (S.R.); giuseppemercuro@gmail.com (G.M.); 2 Department of Clinical and Biological Science, University of Torino, 10043 Obassano (TO), Italy; manuela.aragno@unito.it (M.A.); claudia.penna@unito.it (C.P.); 3 Department of Biomedical Sciences, University of Sassari, 07100 Sassari, Italy; lucia.cugusi@uniss.it; 4 School of Biology, Aristotle University of Thessaloniki, 54124 Thessaloniki, Greece; lazou@bio.auth.gr; 5 Pole of Cardiovascular Research, IREC Institute, UCLouvain, 1200 Brussels, Belgium; christophe.beauloye@uclouvain.be (C.B.); luc.bertrand@uclouvain.be (L.B.); 6 Division of Cardiology, Cliniques Universitaires Saint-Luc, 1200 Brussels, Belgium; 7 Cardiovascular and Metabolic Disorders Program, Duke-National University of Singapore, Singapore 169857, Singapore; d.hausenloy@ucl.ac.uk; 8 National Heart Research Institute Singapore, National Heart Centre Singapore, Singapore 169609, Singapore; 9 Yong Loo Lin School of Medicine, National University Singapore, Singapore 119228, Singapore; 10 The Hatter Cardiovascular Institute, University College London, London WC1E 6HX, UK; 11 Cardiovascular Research Center, College of Medical and Health Sciences, Asia University, Taichung 41354, Taiwan

**Keywords:** diabetic cardiomyopathy, hyperglycemia, ischemia/reperfusion injury, metabolism, mitochondria, remote conditioning, exercise

## Abstract

Metabolic syndrome, diabetes, and ischemic heart disease are among the leading causes of death and disability in Western countries. Diabetic cardiomyopathy is responsible for the most severe signs and symptoms. An important strategy for reducing the incidence of cardiovascular disease is regular exercise. Remote ischemic conditioning has some similarity with exercise and can be induced by short periods of ischemia and reperfusion of a limb, and it can be performed in people who cannot exercise. There is abundant evidence that exercise is beneficial in diabetes and ischemic heart disease, but there is a need to elucidate the specific cardiovascular effects of emerging and unconventional forms of exercise in people with diabetes. In addition, remote ischemic conditioning may be considered among the options to induce beneficial effects in these patients. The characteristics and interactions of diabetes and ischemic heart disease, and the known effects of exercise and remote ischemic conditioning in the presence of metabolic syndrome and diabetes, are analyzed in this brief review.

## 1. Introduction

Diabetic cardiomyopathy and ischemic heart disease are among the leading causes of death and disability in Western countries. Regular exercise can be a strategy for the prevention and treatment of these conditions. Remote ischemic conditioning (RIC), using short periods of ischemia and reperfusion to a limb, due to the similarity with physical exercise, could be an alternative approach in people who cannot exercise. In this brief review, we analyze the characteristics and interactions of these two pathological conditions (diabetic cardiomyopathy and ischemic heart disease) and the known effects of exercise and remote conditioning in the presence of these diseases.

### Diabetic/Metabolic Cardiomyopathy Definition

The term “diabetic cardiomyopathy” was used for the first time in 1972 by Rubler et al. [1]. These authors have described this condition as a myocardial dysfunction in patients with diabetes in the absence of other heart diseases, including coronary artery disease and hypertension. When metabolic disorders related to insulin resistance, obesity, and dyslipidemia are present, we can speak of *metabolic cardiomyopathy* even in the absence of diabetes [2,3]. In fact, diabetes and obesity could induce functional and structural abnormalities in the myocardium independently, suggesting the combination of distinct pathophysiological mechanisms. However, there are overlaps between obesity, insulin resistance, and diabetes states, making it difficult to identify specific mechanisms that lead to myocardial damage. Insulin resistance, the overload of substrates that provide energy and metabolic dysregulation, contributes, at least in part, to this metabolic form of cardiomyopathy [4,5,6].

## 2. Myocardial Features of Diabetic Cardiomyopathy

The common histological characteristic of diabetic cardiomyopathy (DCM) is the presence of interstitial and/or perivascular fibrosis. DCM leads to hypertrophy and apoptosis of cardiomyocytes and is accompanied by increased myocardial oxidative stress. Fibrosis, which is the result of high deposition of extracellular matrix (ECM), leads to myocardial dysfunction, which compromises the relaxation of the left ventricle (LV) and hampers the efficiency of LV contraction. Diastolic LV dysfunction characterized by altered and prolonged ventricular isovolumic relaxation and systolic dysfunction have been confirmed in various experimental models of diabetes mellitus (DM). In fact, heart failure with preserved ejection fraction (HFpEF) is more common in women than in men and is accompanied by evidence of hypertrophy in diabetic patients [7,8]. Thus, diabetic cardiomyopathy includes interstitial fibrosis, cardiomyocyte hypertrophy, and reduced diastolic compliance, which leads to heart failure and especially in patients with both DM and HFpEF.

### 2.1. Molecular Alterations and Myocardial Damage Caused by Hyperglycemia

**Figure 1:**
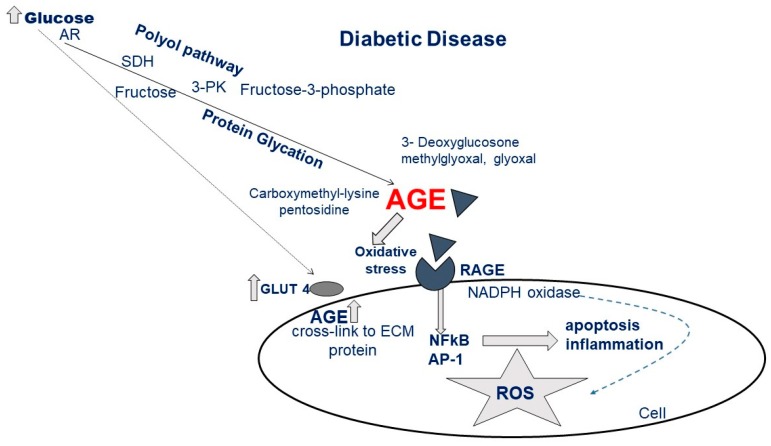
Metabolic pathways leading to advanced glycation end products (AGE) generation and cellular effects after interaction of AGE with the receptor for AGE (RAGE) (AR, aldose reductase; SDH, sorbitol dehydrogenase; 3-PK, 3-phosphokinase; for other acronyms see the list of Abbreviations).

Diabetic cardiomyopathy is the consequence of the activation of multifactorial processes that lead to the damage of myocytes by alterations of numerous molecular pathways. Hyperglycemia is one of the main triggers of these maladaptive processes, including the impairment of insulin signaling, which leads to plasmalemmal depletion of the glucose transporter (GLUT4), changes in free fatty acid (FA) oxidation, protein kinase C (PKC) activation, increased pathways of polyol and hexosamine, and production of advanced glycation end products (AGE) and reactive oxygen species (ROS) (Figure 1). [9,10,11].

**Figure 2:**
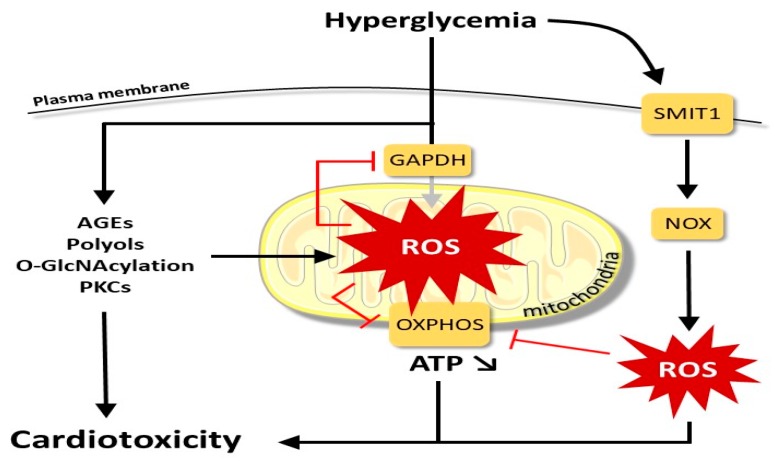
Main factors leading from hyperglycemia to cardiotoxicity (for acronyms see the list of Abbreviations).

In acute and chronic hyperglycemia, a common mechanism in inducing cellular damage is the *increase of ROS generation* in the tissue [12]. An overflow of electrons through the complex chain of electron transport of mitochondria is the main source of ROS under chronic hyperglycemia [12]. However, early generation of high glucose ROS appears to be independent of the mitochondria, and it is attributed to the activation of membrane-bound NADPH oxidase (NOX2) [13]. In fact, acute hyperglycemia is detected by the sodium-myoinositol cotransporter-1 (SMIT1) that promotes NOX2 activation through mechanisms that have yet to be clarified [14]. The hyperglycemia-dependent increase in the polyol pathway reduces the availability of NADPH, increasing oxidative stress [15] (Figure 2).

The hexosamine biosynthesis pathway promotes the protein O-GlcNAcylation, altering important cellular functions, including calcium handling, mitochondrial, and contractile machinery [16,17,18]. The production of endogenous AGE is the result of a non-enzymatic reaction between glucose and proteins and lipids and plays an important role in the pathogenesis of cardiovascular diseases, nephropathy, diabetic retinopathy, and neuropathy, together with the aging process [10,19]. The increase in AGE levels, secondary to hyperglycemia, increases the receptor for AGE (RAGE), resulting in increased ROS production and leading to activation of nuclear factor kappa-light-chain-enhancer of activated B cells (NF-κB) signaling with the production of inflammatory cytokines [20]. Excessive AGE formation leads to the loss of pericytes, increased platelet aggregation, and endothelial dysfunction, all abnormalities that can promote pro-coagulant status with consequent ischemia damage [21].

Classical PKC isoforms are activated by diacylglycerol, which is chronically elevated in hyperglycemia [22]. The activation of PKC isoforms can lead to a series of alterations in cellular signaling both in cardiomyocytes and in endothelial cells, including the activation of NADPH oxidase and the upregulation of Toll-like receptors, TLR-2 and TLR-4, vascular endothelial growth factor (VEGF) activation and inhibition of endothelial nitric oxide synthase (eNOS).

Hyperglycemia and insulin resistance are also associated with the loss of metabolic flexibility, imbalance of lipid metabolism, and exclusive use of FA as an energy substrate. Increased oxidation of FA leads to impaired oxidative phosphorylation and overproduction of ROS mitochondrial decoupling. ROS reacting with NO leads to the formation of reactive nitrogen species (RNS). The increase in FA absorption may exceed the capacity for cardiac use and favor the deposition of triacylglycerol and ceramides, thus leading to cardiomyocyte hypertrophy and steatosis [6,23].

The high glucose level by itself is able to increase the fibrotic processes directly. Indeed, an increase in the deposition of ECM and Transforming Growth Factor beta (TGF-β) upregulation, both involved with a decrease in the activity of the ECM enzyme metalloproteinase, are the main factors in the development of fibrosis. Hyperglycemia can activate the systemic and intra-cardiac renin-angiotensin system with consequent stimulation by angiotensin, of cardiac fibroblasts, and cardiac hypertrophy [24]. Further high levels of ROS in DCM can promote cellular apoptosis directly [25], especially in the presence of an attenuated antioxidant defense system [26,27,28] (Figure 2). The observed increase in pro-inflammatory cytokines in diabetic patients can recruit fibrogenic cells and activate the TGF-β/Smad signal, which in its own right can activate fibroblasts inducing the deposition of ECM [24]. The activation of fibroblasts, in the cardiac interstitium, represents the fundamental basis for the link between metabolic dysfunction and mechanical dysfunction via numerous pathways leading to fibrosis and the activation of remodeling processes [29].

Metabolic alteration, fibrosis, and cardiac remodeling, in general, are common features of hyperglycemia. Cardiac hypertrophy is a structural adaptation in patients with diabetes and represents a predictor of myocardial infarction, stroke, and death [30]. High glucose levels initially induce cardiac tissue hypertrophy as adaptive mechanisms to hemodynamic stimuli often associated with hypertension.

Cardiac hypertrophy may also appear independently of high pressure [31]. Indeed, a greater size of cardiomyocytes has been described by upregulation of hypertrophic gene expression (beta-MHC, type B natriuretic peptide) in hyperglycemic conditions only [32]. Furthermore, a change in the expression of cardiac MHC gene from the alpha-MHC isoform to the beta-MHC isoform and impairments of cardiac myogenic factors, heart autonomic nervous system, and neural crest derivatives (HAND) and myogenic enhancer factor-2 (MEF-2) have all been implicated in pathological cardiac remodeling in response to stress signaling [26].

It is noteworthy that although insulin is known to be a survival hormone, the presence of insulin resistance in diabetes may compromise the protective effect of this hormone. Insulin may protect through activation of the extracellular signal-regulated protein kinases (ERK)1/2, c-Jun NH2-terminal kinases (JNK) MAPK, and phosphatidylinositol 3-kinase (PI3K)/Akt/hexokinase II (HKII) pathway [33,34]. Insulin may lose its cardioprotective effects due to an impairment of MAPK phosphatase-1 expression [33] or because hyperglycemia directly impairs insulin signaling [35].

All in all, these findings suggest that the link between diabetes and metabolic cardiomyopathy are complex and multifactorial and support the increase of myocardial ischemia reperfusion injury (IRI) and the compromised possibility of triggering cardioprotection; two aspects analyzed in the rest of this review (see below).

### 2.2. Diabetes Mellitus and Heart Failure, beyond the Established Risk Factor for Ischemic Heart Disease

**Figure 3:**
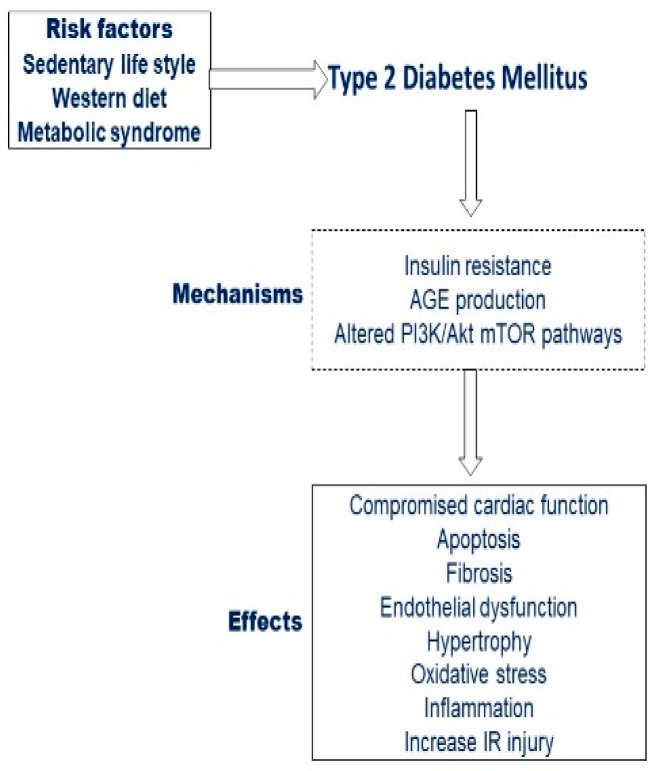
Risk factors leading to diabetes and effects on the cardiovascular system.

DM is closely related to a higher incidence of cardiovascular diseases that include coronary heart disease and a consequent increased risk of cardiovascular death (Figure 3).

The cardiovascular risk in people with diabetes is 2–3 times higher than in those without the disease [36]. A recent prospective study shows that type 1 diabetes mellitus (T1DM) and type 2 diabetes mellitus (T2DM) were strongly associated with an increased risk of cardiovascular disease related to atherosclerosis and post-ischemic heart failure (HF). The cardiovascular risk in people with diabetes is 2–3 times higher than in those without the disease. Furthermore, in the acute environment, diabetes has a dramatic impact on the prognosis of patients with acute coronary syndromes [37].

In addition to coronary heart disease, diabetic patients are at high risk of HF. As it was initially described over 40 years ago by the Framingham cohort, the incidence of HF was 2.4 times higher in men and 5.0 times higher in women with diabetes than in non-diabetics [38]. Based on the most recent data from the Swedish National Diabetes Registry, T2DM patients have also been shown to have an excessively marked risk of HF [39]. In this study, patients (both men and women) with T2DM under the age of 55 and with multiple uncontrolled risk factors had the highest excess risk of HF hospitalization. This excess risk induced by diabetes decreased with age but persisted over time and could be predicted by the presence of a body mass index outside the target range, a low glomerular filtration rate, atrial fibrillation, and a high level of glycated hemoglobin. The risk of HF was slightly lower when glycated hemoglobin levels were within normal values. More interestingly, while HF-associated comorbidities were observed, diabetes was mainly associated with HFpEF, although it could influence the survival of patients suffering from both HF with reduced ejection fraction (HFrEF) and HFpEF [40]. Overall, DM is clearly associated with an excessive risk of HF, regardless of the presence of coronary artery disease.

## 3. Pathophysiology of Hyperglycemia and Diabetes in Cardiac Injury and Dysfunction

As previously exposed, diabetes itself can induce complex cardiac injury. The situation is more complex when acute myocardial ischemia from plaque rupture in a large coronary vessel or acute coronary occlusion overlaps with the presence of DM. Clinically, the perturbations of glucose levels at the time of myocardial ischemia, both hyper and hypoglycemia, have long been associated with poor cardiovascular outcomes. This observation is supported by one of the largest epidemiological studies of its kind, the Cooperative Cardiovascular Project [41]. Surprisingly, this retrospective study of 141,680 patients found that high glucose was, as expected, deleterious in diabetics, but was also highly harmful in patients without known diabetes. Actually, the clinical results in patients without known diabetes were significantly worse than in diabetic subjects, with a steeper relationship between glucose level at the moment of the event and mortality (both at 30-days and 1-year from the event) [41,42]. As summarized elsewhere, this observation has been seen in numerous clinical studies [43], but the challenge has always been to prove causality.

### Effects of Hyperglycemia and Diabetes on Acute Myocardial Infarct Size

Interestingly, when infarct size was measured in patients presenting with AMI, there was a correlation between infarct size—as ascertained by late gadolinium enhancement cardiovascular magnetic resonance and glucose levels at the time of presentation—and infarct size observed in patients without known diabetes compared to diabetics with similar blood glucose levels [44]. Τo ascertain the causality mechanisms observed clinically between glucose and diabetics and infarct size and clinical results, we tried to determine whether the relationship between glucose and myocardial damage could be experimentally modeled.

By definition, diabetic cardiomyopathy is characterized by myocardial dysfunction in the absence of coronary artery disease [1,2,3]. However, it is well known that the presence of diabetes may affect the response to myocardial IRI. In particular, some studies have shown that the diabetic heart is more sensitive to IRI [45,46], while others have suggested that the diabetic heart is less sensitive to ischemic injury [47,48,49]. Therefore, here we briefly consider the effects of diabetes on the response to ischemia/reperfusion, and we separate the pathological effects that can be assigned to the heart itself (intrinsic properties) or changes in the metabolic environment of the circulatory system and the heart. To this aim, we try to summarize the experimental results in acute hyperglycemic conditions, in early and late T1DM and in T2DM in vivo and ex vivo experiments.

Acute hyperglycemic conditions: It seems that acute hyperglycemia promotes vulnerability and increased infarct size of the isolated heart (ex vivo) when glucose is > 30 mM, while greater vulnerability can sometimes already be present for the in vivo condition at glucose levels around 20 mM. This seems counter-intuitive knowing that, in vivo conditions, hyperglycemia will induce increases in the plasma insulin level, thus insulin could act as a protective agent against IRI through the activation of the Akt/hexokinase II (HKII) pathway. However, hyperglycemia directly compromises insulin signaling [34,50,51,52]

Initial T1DM: The initial T1DM induces a cardioprotective state that can be observed in the ex vivo heart. Various mechanisms explain this protected intrinsic state, such as increases in Akt, eNOS, ERK, PKC, mitochondrial HKII, and heat shock proteins. In the in vivo condition, a number of contradictory results are reported. Indeed, in the in vivo condition, all hearts are subject to I/R in the presence of glucose levels > 20 mM. However, exogenous insulin can reduce the acute IRI of the early T1DM heart [33,53,54].

Late T1DM: The reason why protection is lost in the prolonged state of T1DM is not known. This is likely due to prolonged hyperglycemia and hyperosmolarity, chronic low insulin signaling, and/or dyslipidemia.

T2DM: In this condition, most studies report greater vulnerability both in isolated hearts and in vivo. It is likely that insulin-resistance makes the protective Reperfusion Injury Salvage Kinases (RISK) pathway less responsive to the demanding Ischemia/Reperfusion (IR) challenge. The minority of studies reported a protective condition could be related to the obesity paradox and early T2DM, in which insulin signaling may still be effective [55].

Overall, it seems that hyperglycemia may initially induce in the heart a greater resistance to IR challenging, thereafter, especially when DM and insulin resistance develop, IRI is exacerbated.

## 4. Effects of Remote Conditioning Protocols in Diabetes

**Figure 4:**
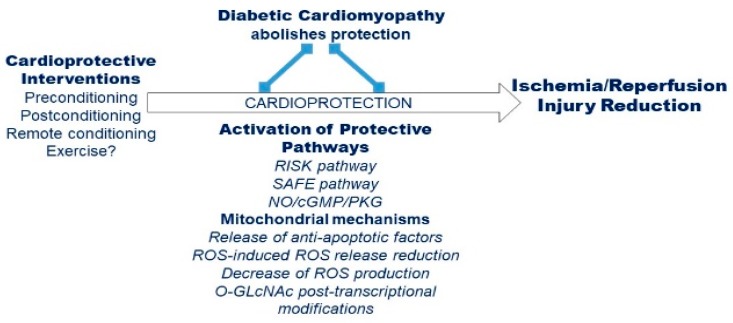
Overview of cardioprotective mechanisms and pathways altered/inhibited by diabetic condition.

Remote ischemic conditioning (RIC) has become the most popular form of mechanical cardioprotection. It is a safe, non-invasive procedure, which avoids manipulation of the occluded coronary lesion, as classical preconditioning requires. RIC has been used in surgical coronary revascularization and elective percutaneous coronary intervention [56]. Numerous research groups have shown the activation of an endogenous protective adaptive response by RIC. In preconditioning RIC (pre-RIC), short cycles of IR are delivered to the remote organ before index ischemia, while preconditioning RIC (per-RIC) is characterized by short IR cycles to the remote organ during the time of sustained cardiac ischemia. In postconditioning RIC (post-RIC), the short cycles of IR are delivered to the remote organ during the heart reperfusion [57,58,59]. Numerous studies have reported the efficacy of RIC in patients undergoing heart surgery and the coronary artery disease [60]. Several different *cardioprotective mechanisms* have been described for RIC [60,61,62,63]. It seems that the three forms of RIC have similar mechanisms (Figure 4). Some of these mechanisms may be in common with exercise-induced cardioprotection (see below).

An interesting aspect relative to the cardioprotective modalities of RIC is how it is conveyed to the distant organ. Indeed, neural and humoral factors play a role in RIC [64,65]. Among the various possibilities, the *neural pathway* is particularly interesting in diabetic conditions [65,66].

Indeed, RIC-protection is abolished in the diabetic animal model, most likely due to the presence of diabetic neuropathy, reducing the functionality of the neural pathway [66,67]. The main mechanisms on how diabetes may impair the cardioprotective effects of RIC have been recently summarized [60]. For instance, the impairment of the cardioprotective humoral factor may contribute in part to attenuating RIC-induced cardioprotection [60]. In addition, humoral impairment has been linked to diabetic neuropathy [67]. Although it is not easy to separate in patients under sulphonylurea treatment the drug effect from that of diabetes, it is noteworthy that cardioprotection by RIC is lost in sulphonylurea-treated diabetic patients undergoing coronary revascularization [68]. The RISK (PI3K/Akt/eNOS) and the Survivor Activating Factor Enhancement (SAFE, JAK-STAT3) pathways within the myocardium are important protective signaling pathways involved in RIC protection. A marked inhibition of the SAFE (JAK-STAT3) and RISK (PI3K/Akt/eNOS) signaling cascades in the presence of diabetes have been reported [69,70,71]. RIC involves also a signaling through the SDF-1α/CXCR4 signaling axis [72].

Actually, it has been suggested that PTEN/Akt pathway is altered in the presence of diabetes [73]. However, results suggest that diabetes does not impair the functioning or activation process of RISK pathway, as the activation/phosphorylation of kinases of RISK was common either in the healthy and diabetic hearts rats to a comparable degree [60]. All in all, it is likely that a stronger stimulus is required in diabetic conditions to activate RISK elements. For instance, exogenous administration of protective hydrogen sulfide (H_2_S) donor confers cardioprotection in diabetic rats by activation of endogenous protective RISK pathway [61,63,74]. An important post-translational modification of proteins dependent by changes of the extracellular concentration of glucose is the *O-Linked β-N-acetylglucosamine (O-GlcNAc) glycosylation* [75]. Various protective strategies, including RIC, increase the levels of O-GlcNAc. However, in the diabetic model, the levels of O-GlcNAc cannot increase because levels are already high before RIC stimulus [75]. It has been reported that RIC down-regulates mTOR [76]. It seems that RIC induced cardioprotection is abolished during acute hyperglycemia because of the activation of *mTOR-mediated insulin negative feedback loop* in diabetic conditions [77,78]. In the diabetic model, the pre-treatment with rapamycin induces an improvement of the cardiac function [78,79].

In several clinical studies evaluating the efficacy of RIC in terms of levels of marker of ischemic cell death, either beneficial [80,81] or no effect have been shown [82,83,84]. These different outcomes may be due to patient populations with various comorbidities such as diabetes. Indeed, the beneficial effects of RIC in humans is influenced by various factors including aging [85,86,87], cardiovascular comorbidities (e.g., angina pectoris, heart failure, cardiac hypertrophy, hypertension), and/or metabolic diseases (e.g., insulin resistance and diabetes mellitus) [56,59,88,89,90]. In addition, the target organ may be differently influenced by diabetic conditions, as shown in a double-blinded, placebo-controlled, multicenter, randomized study in humans designed to analyze RIC effect on contrast-induced nephropathy during the percutaneous coronary intervention (PCI) (as defined from serum creatinine) [91]. These authors have shown that the protective effects of RIC against contrast-induced damage was lost in diabetic patients. However, and strangely enough, they reported a beneficial reduction of periprocedural myocardial infarct (PMI, as defined from CK-MB or troponin) in diabetic but not in non-diabetic patients. The authors recognized that this study was under-powered for analyzing the PCI-induced PMI [92]. However, studies designed to evaluate the effect of diabetes on RIC specifically are scarce, and more studies are required to understand the effect of diabetes on the protective effect of RIC [93]

## 5. Type 1 and 2 Diabetes Mellitus and the Cardiovascular Regulation during Exercise

**Figure 5:**
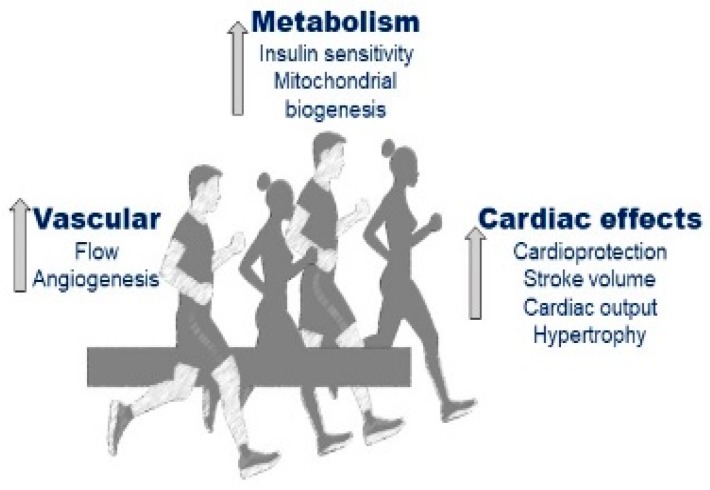
Putative effects of exercise on the cardiovascular system.

Exercise training is beneficial for patients suffering from both T1DM and T2DM as it reduces cardiovascular risk (Figure 5).

However, it should be considered that impairments in cardiovascular regulation may develop in both types of DM and that these patients may suffer from several cardiovascular abnormalities in response to effort [94]. In healthy individuals, the circulatory adjustment to exercise is the result of a hemodynamic regulation deriving from the activity of cardiovascular control centers located in the *Medulla Oblongata.* Specifically, these centers adjust sympathetic and parasympathetic tone proportionally to different feedback: The neural drive from the motor cortex, the metabolic and mechanical status of the contracting muscle, and the blood pressure level [95]. The resulting autonomic modulation aims to guarantee a sufficient blood delivery and wash-out of metabolic end-products in the contracting muscle and to maintain the target level of blood pressure. This autonomic activity is highly effective, and it finely adjusts hemodynamics so that normal individuals safely exercise without experiencing excessive variation in blood pressure [96].

However, as both type 1 and 2 DM affect autonomic activity, this fine cardiovascular regulation can be disrupted. For instance, insulin by-itself may affect cardiovascular regulation as it increases sympathetic tone by exerting centrally-mediated sympathetic stimulation; moreover, insulin exerts vasodilatory effects [94,97].

### 5.1. Cardiovascular Dysregulation in T1DM

In T1DM, a reduction in catecholamine levels in response to effort has been observed. This phenomenon has been associated with reduced sensitivity to catecholamines of some tissues, such as the medulla glands and myocardium. Thus, in these patients, signs, and symptoms of sympathetic deficit may develop during exercise and recovery [98,99]. A possible explanation for the described sympathetic deficit is that repeated episodes of subclinical hypoglycemia continuously stimulate the sympathetic tone. In the longer term, this reduces the responsiveness of the sympathetic system and limits its capacity to properly adjust hemodynamics during exercise [100]. It is to be underscored that this reduced capacity to recruit sympathetic activity seems to be well tolerated in young individuals. Indeed, the exercise tolerance of young T1DM subjects is in normal limits, even in the presence of autonomic failure [98,99,100].

Furthermore, several other hemodynamic deficits in response to exercise have been described in T1DM. Specifically, reductions in maximum heart rate (HR), stroke volume (SV), and diastolic function have all been demonstrated. Other hemodynamic abnormalities are the defect in peripheral vascular responsiveness and the reduction in circulating blood volume [98,101,102], which together concur in reducing the SV response and in limiting the capacity to modulate systemic vascular resistance (SVR). All these phenomena may hamper the capacity to properly adjust cardiac output (CO) and blood pressure, thereby impairing muscle blood flow during effort.

In summary, in patients suffering from T1DM a dysregulation in central and peripheral hemodynamics during exercise has been often reported, with lower HR, SV, and CO in comparison with healthy individuals; moreover, the capacity to vasoconstrict the arteriolar bed can be reduced. All these hemodynamic deficits affect blood pressure regulation during exercise, with reduced levels in comparison with normal subjects.

### 5.2. Cardiovascular Dysregulation in T2DM

There is convincing evidence that in T2DM chronic hyperinsulinemia induces sympathetic overactivation. Moreover, other investigations found that sympathetic activity was exaggerated in obesity and that this phenomenon can be counteracted by weight lost. Yet, reduced parasympathetic activity has been demonstrated in individuals with obesity [103]. Overall, these findings are in line with the concept that there is a common link among obesity, autonomic dysfunction, hyperinsulinemia, and T2DM.

It is then not surprising that differently from what has been found in T1DM, T2DM, and insulin resistance have been often associated with an increase in sympathetic tone [104]. This may explain why these subjects often experience elevated blood pressure and exaggerated increments in SVR in response to effort [105,106]. Moreover, cerebral auto-regulation has been found impaired in these patients [106]. Although the precise mechanism(s) responsible for this vascular dysfunction has not been completely elucidated, several clues suggest that autonomic abnormalities may play a key role [103,107,108]. Specifically, it was proposed that insulin resistance leads to an imbalance between NO and endothelin-1 (ET-1) production, with the effects of ET-1 (vasoconstriction) prevailing over NO-induced vasodilation [109].

Similar to what has been reported for T1DM, a reduction in blood volume has also been demonstrated for T2DM [110], and this, together with a systolic deficit, may impair cardiac preload and SV response to exercise in these patients [111].

In summary, in patients suffering from insulin resistance and T2DM, a tendency towards arteriolar constriction, exaggerated SVR, and elevated blood pressure in response to exercise have been demonstrated. Moreover, a reduced SV in response to exertion has also been observed.

In summary, patients suffering from type 1 and 2 DM may experience different challenges in hemodynamic regulation during exercise. Elucidation of the cardiovascular abnormalities and dysregulation need further research. For instance, it remains to be demonstrated that the sympathetic deficit present in T1DM exerts negative effects on the hemodynamic response to effort.

Moreover, the precise origin of the sympathetic over activation shown by patients with T2DM is still unclear. Similarly, the origin of the exaggerated arteriolar constriction is still to be fully elucidated. The precise role played by the reduction in NO production and the NO/ET-1 imbalance has not been definitively ascertained yet. Very likely other metabolites still to be discovered may play a role in the phenomenon.

Finally, in both types of DM, it is still to be clarified the role played by the reduced circulating blood volume in the blunted SV response during exercise.

All the described cardiovascular abnormalities may limit exercise tolerance, with a negative impact on the quality of life of these patients.

## 6. Exercise Training for the Management of Diabetes Mellitus: Classic Approaches

Exercise has been demonstrated to be a valuable therapeutic option for individuals diagnosed with type 1 and 2 DM. It has been described that the risk for cardiovascular diseases is increased in these patients, with approximately a two-four-fold higher risk as compared to healthy subjects [112]. Several scientific reports in people with DM have reported the beneficial effects of interventions based on conventional forms of exercise, mainly consisting of aerobic and strength training programs [113]. Based on this body of evidence, current guidelines recommend people with DM to train for no less than 150 min per week at moderate to vigorous intensity, with a frequency of at least 3 days/week [113]. For its part, the European Association of Preventive Cardiology Exercise Prescription in Everyday Practice and Rehabilitative Training (EXPERT) recommends a daily physical activity of at least 30 min at moderate intensity to improve cardiac function and glycemic control in selected subjects with DM and impaired myocardial function [114].

More than 80% of T2DM are patients suffering from metabolic syndrome (MS). This condition is characterized by chronic inflammation, obesity, dyslipidemia, hypertension, and chronic hyperglycemia with insulin resistance [115]. Of note, a chronic low-grade inflammation can be considered a common denominator of both MS and T2DM [116]. Subjects with insulin resistance and T2DM are characterized by impaired insulin action and body glucose uptake in the skeletal muscle. Exercise training enhances skeletal muscle glucose uptake by rapidly increasing the expression of GLUT-4 mRNA in the skeletal muscle [117,118]. This effect is achieved due to insulin-dependent and insulin-independent mechanisms [119,120]. Although the precise signaling mechanisms that mediate the exercise-induced increase in glucose transport are still not completely understood, it has been suggested that exercise activates multiple signaling pathways. Among others, the adenosine monophosphate (AMP)-activated protein kinase, its upstream kinases LKB1 and the Ca^2+^/Calmodulin-dependent protein kinase-β, but also other Ca^2+^/Calmodulin-dependent protein kinases may be involved in this phenomenon, with some degree of redundancy among them. Exercise also increases insulin effectiveness in stimulating glucose transport, and this effect has been observed up to 48 h after exercise bouts in humans, although the mechanisms mediating this effect are still unknown [121].

Exercise training has been shown to decrease the circulating level of inflammatory mediators producing an anti-inflammatory effect [122]. Moreover, habitual exercise promotes a decrease in body weight and visceral fat accumulation, an increase in high-density lipoprotein cholesterol, a reduction in mean blood pressure level, and an improvement in insulin sensitivity [123,124].

Many studies have investigated the role of different training variables, such as duration, intensity, frequency, on T2DM, especially on glycated hemoglobin reduction. In a meta-analysis comparing patients with T2DM and controls, the effects of combined aerobic exercise training (AET) and resistance training (RT) have been investigated. It was reported that the combination of RT with AET exerted more beneficial effects on glycemic control than AET alone [125]. In fact, RT enhanced insulin sensitivity by increasing GLUT-4 transporter expression in the adipose tissue and in the skeletal muscle in the presence of adequate beta-cell function [126].

Another recent investigation conducted in T2DM patients compared the effect of AET, lasting at least 30 min per day from 3 to 7 days a week, with RT [127]. It was observed that RT improved muscle strength by 10%–15% and exerted beneficial effects on lipid profile, blood pressure, bone mineral density, insulin sensitivity, and muscle mass [128,129]. Balducci et al. [130] reported that the combination of AET and RT improved glycated hemoglobin in addition to global improvements in cardiovascular risk factors in these patients. Other research demonstrated that combined training produced a greater improvement in glycated hemoglobin than AET or RT alone [131]. High intensity interval training, comprising short repeated bouts of maximal effort (lasting 30 s) alternated with brief periods of rest (from 30 to 60 s), has been demonstrated to increase skeletal muscle oxidative capacity, glycemic control, and insulin sensitivity in adults suffering from T2DM [132].

In contrast to T2DM, the management of T1DM still remains a challenge as these patients may show disproportionate exercise intolerance, which may contribute to a poor exercise training adherence [133,134]. Nevertheless, physical activity may play an important role in the treatment of subjects with T1DM. It has been demonstrated that the amount of time spent in non-exercise physical activity, which includes daily activities such as going to work and school, washing clothes, cleaning the floor, etc., positively affects daily glucose excursions in these patients [135].

During AET, insulin secretion decreases, while glucagon secretion increases in the portal vein in order to release glucose from the liver to support the working muscles [136,137]. Trained subjects with T1DM demonstrated a greater reduction in blood glucose concentrations during aerobic exercise rather than untrained patients [138]. High-intensity interval training induced an increased oxidative capacity of skeletal muscle and blunts the rates of glucagon breakdown, which could be a possible protective effect against hypoglycemia after exercise [139]. However, a recent study published by Štotl et al. [140], conducted in a cohort of 109 T1DM patients, demonstrated that physical activity in leisure time did not affect glycemic control measured and glycated hemoglobin in T1DM individuals.

In summary, for patients with both type 1 and 2 DM, 150 min of weekly physical activity is recommended with no more than two consecutive days of rest. RT is also recommended two or three times a week [128]. Regular exercise should be encouraged and supported by health care because it appears to have a positive effect on the reduction of cardiovascular and metabolic risk in these subjects.

## 7. Exercise as Therapy for Diabetes Mellitus: Unconventional and Novel Exercise-Based Approaches

Recent investigations in people with DM and cardiovascular disease have reported beneficial effects following unconventional forms of exercise, such as aquatic-based exercise, Nordic walking, specific gym-fitness activities, Yoga, Pilates, Tai Chi, and dance-based activities. Less common approaches have been shown to induce positive cardiovascular effects, also improving general well-being, social inclusion, and quality of life of the participants [141,142,143,144,145,146,147].

From a biopsychosocial standpoint, these complementary and/or alternative exercise approaches are mainly focused on the relationships between brain, body, and behavior and their effects on health and disease, with the common awareness that a positive attitude of the mind may be able to promote overall physical and mental health and well-being. From a physiological point of view, beneficial cardiovascular effects following aquatic/swimming training were reported in the animal model. A swimming training program of 90 min per day, 5 days a week performed for 8 weeks proved to be effective in attenuating myocardial fibrosis and contractile dysfunction [148], reduced cardiac glycogen storage [149] and level of tumor necrosis factor-α (TNF-α), and increased capillary density [150] in Wistar rats with streptozotocin-induced DM. Overall, in these rats without antidiabetic drug therapy, an 8-week swimming workout was able to mitigate the vast majority of the pathological changes related to DM.

In humans with DM, 12 weeks of cycling training performed in warm water achieved similar improvements to those of land-based cycling exercise in plasma nitric oxide concentrations, arterial stiffness, and flow-mediated dilation in the popliteal artery; on the other hand, a significant improvement in the microvascular reactivity indices was found only in the aquatic exercise group [151]. In line with these findings, a 12-week aquatic-based exercise program was able to improve diastolic function in men diagnosed with DM, detected by the significant reduction in the E/E’ echocardiographic ratio (i.e., the ratio of the peak early mitral inflow velocity over the early diastolic mitral annular velocity) [152]. This evidence may be of particular relevance when considering that diastolic dysfunction represents one of the first signs of diabetic cardiomyopathy. Ring et al. [153] reported that a 4-month Nordic walking training was not sufficient to improve vascular function (i.e., aortic pulse wave velocity, aortic augmentation index, arterial stiffness, and reflection index and systemic vascular resistance). In contrast, Fiodorenko-Dumas and colleagues reported a significant reduction in serum levels of von Willebrand factor—a useful marker for endothelial cell efficiency—after 6 weeks of Nordic walking training, likely suggesting a positive influence of such unconventional outdoor activity on vascular function [154]. When considering gym-based activities, Zumba fitness and mini-trampoline rebounding exercise emerged as the most investigated ones [142,144].

Overall, these forms of fitness workout have reported beneficial effects on common metabolic and anthropometric outcomes (e.g., body weight, body fat percentage, insulin resistance) [142,144,155], although little or no attention was paid to evaluating specific cardiovascular parameters related to peripheral and central vascular function. With regard to mind-body exercise therapies, Borges and colleagues [156] showed that 4 months of a dance-based exercise program reduced plasma inflammatory markers and acute-phase proteins, and increased the concentration of anti-inflammatory cytokines in individuals with DM. In addition, Melo et al. reported that 12 weeks of Pilates induced significant improvements in glycemic control in older women with DM [157]. It is noteworthy that most studies on alternative mind-body exercise therapies have focused on Yoga and Tai Chi, highlighting their positive effects on DM management. Indeed, recent summaries of the literature concluded that both Yoga and Tai Chi interventions are highly feasible and suitable for people with DM, also allowing improvements in glycemic control, cardiovascular risk factors, and biopsychosocial outcomes [146,147].

In summary, while the body of evidence accumulated so far seems promising, further high-quality research is needed to elucidate the specific cardiovascular effects of these emerging and unconventional forms of exercise in people with DM. In particular, studies must ascertain the long-term effects on heart function, as well as the association between participating in such exercise programs and their potential protective role against the risk to develop peripheral and central vascular DM-induced complications.

## 8. Conclusions and Future and Recommendations for Future Research

On the basis of the scientific literature, clinicians should promote exercise as a valid strategy to counteract adverse metabolic and cardiovascular effects of both T1DM and T2DM. Remote ischemic conditioning shares similar mechanisms with exercise-induced cardioprotection. However, its potential role in protecting the myocardium in patients with DM is largely unknown. Studies specifically designed to understand the potential protective effect of RIC on T1DM and T2DM DM are warranted.

Although exercise is a well-established tool to manage both types of diabetes, some aspects related to cardiovascular regulation during exercise need further research. For instance, it is still not clear the origin of the impaired endothelium-induced vasodilation.

A further point to be clarified is which type of exercise should be recommended. Apart from classical aerobic training programs, other kinds of training have been proposed. However, their safety and applicability still require a full investigation. Moreover, future study is required to define the proper intensity, duration, and frequency of exercise for prevention of cardiac events in these patients.

## Abbreviations

3-PK	phosphokinase
AET	aerobic exercise training
AGE	advanced glycation end-products
AR	aldose reductase
beta-MHC	beta-myosin heavy chain
BNP	type B natriuretic peptide
CK-MB	creatine kinase myocardial band
CO	cardiac output
DCM	diabetic cardiomyopathy
DM	diabetes mellitus
ECM	extracellular matrix
eNOS	endothelial nitric oxide synthase
ERK	extracellular signal-regulated kinases
ET-1	endothelin-1
FA	fatty acids
GLUT-4	Glucose transporter type 4
HAND	heart autonomic nervous system and neural crest derivatives
HbA1c	Hemoglobin A1c
HF	heart failure
HFpEF	heart failure with preserved ejection fraction
HFpEF	heart failure with reduced ejection fraction
HKII	hexokinase II
HR	heart rate
IR	Ischemia/Reperfusion
IRI	Ischemia Reperfusion Injury
JAK	Janus kinase
LV	left ventricle
MEF-2	myogenic enhancer factor-2
MS	metabolic syndrome
mTOR	mammalian target of rapamycin
NADPH	nicotinamide adenine dinucleotide phosphate
NF-κB	nuclear factor kappa-light-chain-enhancer of activated B cells
NO	nitric oxide
NOX2	NADPH oxidase 2
O-GlcNAc	O-Linked β-N-acetylglucosamine
PCI	percutaneous coronary intervention
per-RIC	perconditioning RIC
PKC	protein kinase C
PMI	periprocedural myocardial infarct
post-RIC	postconditioning RIC
pre-RIC	preconditioning RIC
PTEN	Phosphatase and tensin homolog deleted on chromosome 10
RAGE	receptor for AGE
RIC	Remote ischemic conditioning
RISK	Reperfusion Injury Salvage Kinases
RNS	reactive nitrogen species
ROS	reactive oxygen species
RT	resistance training
SAFE	Survivor Activating Factor Enhancement
SDH	sorbitol dehydrogenase
Smad	small mother against decapentaplegic
SMIT1	sodium/myo-inositol cotransporter-1
STAT-3	signal transducer and activator of transcription 3
SV	stroke volume
SVR	systemic vascular resistance
T1DM	type 1 diabetes mellitus
T2DM	type 2 diabetes mellitus
TGF-β	Transforming growth factor beta
TLR	Toll-like receptor
TNF-α	Tumor necrosis factor-α
VEGF	vascular endothelial growth factor
